# Optimization of Punch Shaft Design for Reduced Punching Force and Enhanced Tool Life in S500MC Steel Sheet Forming

**DOI:** 10.3390/ma19071470

**Published:** 2026-04-07

**Authors:** Abdelwaheb Zeidi, Khaled Elleuch, Şaban Hakan Atapek, Jarosław Konieczny, Krzysztof Labisz, Janusz Ćwiek

**Affiliations:** 1 Materials Engineering and Environment Laboratory (LGME), National School of Engineers of Sfax (ENIS), University of Sfax, Sfax 1173-3038, Tunisia; abdelwaheb.zeidi@gmail.com (A.Z.);; 2 Laboratory of High Temperature Materials, Department of Metallurgical and Materials Engineering, Kocaeli University, 41001 İzmit, Türkiye; hatapek@kocaeli.edu.tr; 3 Department of Railway Transport, Faculty of Transport and Aviation Engineering, Silesian University of Technology, 40-019 Katowice, Poland; krzysztof.labisz@polsl.pl (K.L.); janusz.cwiek@polsl.pl (J.Ć.)

**Keywords:** punching, velocity, stress, AISI D2, Johnson Cook

## Abstract

This study presents a comprehensive numerical and experimental investigation into the influence of punch shaft geometry on punching force and tool durability in the cold forming of S500MC steel sheets using an AISI D2 punch. Finite element analyses were conducted to evaluate the effects of varying punch shaft diameters on stress distribution, deformation behavior, and resultant punching forces. Experimental validation was performed through controlled punching tests, measuring force responses and assessing tool wear. The results demonstrate that optimizing the punch shaft diameter reduces the maximum punching force and minimizes stress concentrations, thereby enhancing tool life. Specifically, larger punch shaft diameters contribute to more uniform stress distribution and decreased risk of premature tool failure. These findings provide valuable insights for tooling design in high-strength steel sheet forming processes, enabling improved efficiency and cost-effectiveness in manufacturing operations.

## 1. Introduction

Despite ongoing industrial advancements and technological innovations, the cold punching process remains a complex manufacturing operation fraught with persistent challenges that significantly affect productivity and operational efficiency [1]. One of the most critical issues faced by practitioners and researchers alike is the premature wear and failure of punching tools [2]. Tool wear not only increases maintenance and replacement costs but also deteriorates product quality and consistency, ultimately impeding the scalability and economic viability of mass production systems [3]. These challenges underscore the necessity for a deeper scientific understanding of the cold punching process and the development of optimized operational parameters that can simultaneously enhance tool durability and maintain high-quality output [4].

Cold punching is an essential technique widely employed in metal forming industries for fabricating components with intricate geometries and tight tolerances without the need for heating the material [5]. This process is favored due to its advantages such as improved mechanical properties of the finished product, reduced energy consumption, and enhanced dimensional accuracy [6]. However, the process involves complex interactions between the workpiece material, tooling geometry, and process parameters, which collectively influence the stresses, strains, and thermal effects experienced by the tooling system [7]. Consequently, the optimization of punching parameters such as punch velocity [8], clearance [9], lubrication conditions [10], and material properties [11] has become a focal point for research aimed at mitigating tool wear and improving process efficiency [12].

Several studies have contributed valuable insights into the cold punching and cold forming domains. Altamirano-Fortoul et al. [12] highlighted that cost-efficiency in product manufacturing can be significantly improved by adhering to an optimal machining range and carefully selecting punching parameters, along with minimizing raw material costs. This study emphasizes the economic implications of process parameter optimization beyond tool longevity, demonstrating an integrated approach to cost reduction [13]. Bhattacharyya et al. [13] conducted experimental investigations on cold roll forming, focusing on the effects of roll velocity and roll axisymmetric impact on punching forces. Their work provided a scientific basis for understanding process mechanics, though it was limited by consideration of only two parameters and was restricted to the cold roll forming context [14]. This gap points to the need for a more comprehensive examination of multifactor interactions and their cumulative impact on tool performance and product quality [15]. Expanding on material behavior under forming conditions, Lindgren utilized finite element analysis (FEA) to study the effects of bending angle and material strength on strain distribution and deformation length. The results indicated that increasing material yield strength reduces peak longitudinal strain and extends deformation length, implying that material properties play a pivotal role in determining the mechanical response of the workpiece and the associated tooling load during punching [16]. Such numerical simulations provide crucial predictive capabilities that can guide the design of both tools and process parameters [17]. Hong et al. [8] introduced a numerical simulation program tailored for cold roll forming processes, examining factors such as sheet thickness, roll diameter, roll velocity, and material characteristics to predict forming length. However, their study acknowledged limitations, particularly the omission of punch-die clearance and lubrication effects, which are known to substantially influence punching force and tool wear. This highlights a critical area for further research to develop more holistic models that account for these variables [18]. Bui and Ponthot [17] advanced the understanding of cold roll forming by employing three-dimensional finite element simulations to investigate the influence of friction, roll velocity, stand distance, and material properties. Their findings revealed a strong dependency of product quality on material yield limit and work hardening exponent, illustrating how intricate material behavior under process conditions critically affects outcomes. This study underscores the importance of integrating detailed material modeling into process optimization efforts [19]. Further advancing parameter optimization, Qazani et al. [18] applied the response surface methodology (RSM) to optimize cold rolling by minimizing the spring-back angle, an important factor affecting dimensional accuracy of rolled sections. Their work demonstrated that careful design of roll profiles via optimization techniques can yield defect-free products [20]. This approach exemplifies how statistical and computational methods can be effectively combined to refine manufacturing processes.

Building upon these prior contributions, the present study introduces novel design solutions facilitated by advanced three-dimensional (3D) modeling software, including modifications to shaft geometry, to explore their effects on cold punching performance [21]. Numerical simulations using the Finite Element Method (FEM) are conducted to analyze the distribution of Von Mises stress within the tooling and workpiece [22]. Additionally, response surface methodology is employed to systematically investigate the impact of key punching parameters, such as punch velocity, on the maximum punching force [23]. This integrated experimental-numerical approach aims to not only elucidate the underlying mechanics of cold punching but also to provide practical guidelines for parameter selection that optimize tool lifespan, improve product quality, and reduce manufacturing costs [24]. In summary, the continuing demand for efficient, cost-effective, and high-quality cold forming processes necessitates comprehensive research efforts that combine experimental investigations, numerical simulations, and optimization techniques. This study advances the field of cold punching technology by systematically addressing the multifaceted interactions between tooling design, material behavior, and processing parameters. Through comprehensive analysis, we introduce innovative solutions to overcome the challenges that currently limit large-scale industrial applications. Specifically, this study introduces a novel, multi-faceted approach to cold punching technology by rigorously evaluating and comparing alternative tooling designs—specifically the double-shear punch and staircase shaft design—to address critical industrial challenges. Through a comprehensive comparative analysis, we assess the impact of these designs on punching efficiency, wear resistance, and operational stability, establishing a robust framework for selecting optimal configurations tailored to diverse industrial requirements. The double-shear punch demonstrates clear advantages, including a significant reduction in punching force and enhanced tool longevity, while minimizing material deformation and improving the precision of punched components. Meanwhile, the staircase shaft design introduces an innovative solution for optimizing load distribution and mitigating stress concentrations, thereby enhancing tool durability and operational reliability—particularly in high-volume production environments. Together, these advancements represent a holistic improvement in cold punching technology, offering scalable and sustainable solutions for industrial applications.

A wide range of experimental investigations and studies have been conducted with the objective of identifying optimal parameters to effectively reduce punching force in metal forming processes [25]. Despite the extensive research efforts, the findings reported in the literature exhibit considerable variation, often with significant discrepancies between the results of different studies [26]. This variability can primarily be attributed to differences in experimental setups, material properties, tooling configurations, and process conditions [27]. Consequently, each experiment tends to yield its own specific set of parameters and outcomes, reflecting the complex and multifactorial nature of the punching process.

## 2. Materials and Methods

### 2.1. General Context and Problematic Aspects

In this context, the present study focuses on the performance of an AISI D2 tool steel (Chafik Loukil establishment, Sfax, Tunisia) punch during the punching of a sheet metal known commercially as metrix head key which corresponds to the S500MC (Chafik Loukil establishment, Sfax, Tunisia) grade steel. The punching operation involves the creation of a hole with a diameter of 3 mm in a metal sheet whose thickness matches the punch diameter. This setup is representative of precision punching applications where the interplay between tool geometry, material properties, and process parameters critically influences both the punching force and tool life. The AISI D2 punch is subjected to complex mechanical stresses during the operation, including both compressive loads and buckling phenomena. These mechanical challenges arise due to the high localized stresses at the punch tip as it shears through the metal sheet, combined with the slender geometry of the punch that predisposes it to potential stability issues under axial loading. Understanding the stress state and deformation behavior of the punch is essential to optimize its design and operational parameters to mitigate premature failure and enhance durability. The mechanical and metallurgical properties of the materials involved play a pivotal role in the punching process. Table 1 summarizes the key mechanical characteristics of the AISI D2 tool steel and the S500MC sheet metal. AISI D2 is classified as a high-carbon, high-chromium tool steel, renowned for its exceptional wear resistance and high hardness [28]. These properties are primarily attributed to its unique alloy composition and heat treatment, which result in a hardened microstructure capable of withstanding abrasive and adhesive wear mechanisms commonly encountered during punching operations [29]. The superior wear resistance of AISI D2 makes it a preferred choice for tooling applications where prolonged service life and dimensional stability are critical [30]. Conversely, the S500MC steel, a low alloy structural steel, is characterized by its high formability and consistent quality, which are essential for reliable and accurate forming operations [31]. The alloying elements in S500MC steel contribute to a balanced combination of mechanical properties, such as sufficient strength and ductility. This enables the material to undergo plastic deformation during punching without cracking or excessive work hardening [32]. This combination of properties makes S500MC well suited for industrial applications requiring complex shaping and high dimensional fidelity [33].

The interaction between the AISI D2 punch and the S500MC sheet during the punching process thus represents a technically demanding scenario where the mechanical properties of both materials must be carefully considered to achieve optimal performance. The high hardness and wear resistance of the punch material ensures tool longevity, while the favorable formability of the sheet metal reduces the punching force required and minimizes the risk of defects in the finished hole [34]. Overall, the experimental results substantiate the suitability of AISI D2 steel as a punching tool material for use with S500MC sheets under the defined operating conditions. The provided results indicate that the mechanical properties of S500MC steel are significantly influenced by its alloying composition, as evidenced by the observed balance of strength and ductility during deformation processes. Continued investigation incorporating numerical simulations and advanced characterization techniques is recommended to deepen understanding of the complex interactions at play and to support the development of predictive models for process optimization. Increasing the lifespan of punching tools remains a significant and ongoing challenge for mold makers and manufacturing engineers. The durability of these tools is in accordance with the forces exerted during the shaping process, where the primary objective is to minimize the maximum shaping force—particularly the punching force in this context.

**Figure 1:**
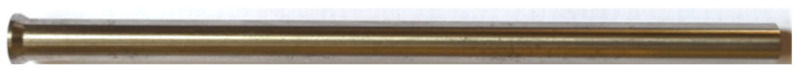
Industrial tool used in metrix head key punching.

Excessive punching forces not only accelerate tool wear but also increase the likelihood of mechanical failure, thereby limiting the tool’s effective service life and negatively impacting production efficiency, especially in high-volume manufacturing environments [35]. The punching tool commonly employed in industrial applications typically features a cylindrical geometry, as illustrated in Figure 1. This design, while practical for various punching operations, is susceptible to a range of damage mechanisms that manifest on both the punch shaft and its head. Empirical observations and studies, such as those reported in [36], have documented several prevalent forms of damage that compromise tool integrity. These include chipping, which involves the detachment of small fragments from the punch surface; cracking, which represents the initiation and propagation of fractures under cyclic loading; and swelling or plastic deformation of the punch head due to localized stresses exceeding the material’s yield strength [37]. Such damage not only degrades the tool’s dimensional accuracy but also poses risks of catastrophic failure if left unaddressed. The specific dimensions of the punching tool under consideration further influence its mechanical behavior and susceptibility to failure [38]. The punch head, responsible for direct contact and shearing of the sheet metal, has a diameter of 4 mm, while the punch shaft diameter is 3 mm with an overall length of 50 mm. This slender shaft geometry, combined with the relatively small head size, contributes to complex stress distributions during operation, including compressive, bending, and buckling stresses. The mechanical demands placed on the punch are substantial, with punching forces reaching magnitudes on the order of 1800 MPa. Such high stress levels are primarily induced by the resistance of the workpiece material to deformation and fracture during hole formation. The elevated punching force has two critical consequences. First, it accelerates wear and fatigue damage on the punch, thereby shortening its operational lifespan and increasing downtime and tool replacement costs. Second, the high force requirement contradicts the demands of large-scale series production, where rapid throughput, tool reliability, and minimal maintenance are essential. The resultant trade-off between tool durability and production efficiency presents a substantial engineering challenge. To address these issues, research and development efforts are increasingly focused on optimizing tool geometry, material selection, and process parameters to reduce punching force without compromising product quality. Strategies such as refining punch head shape, adjusting clearance between punch and die, improving lubrication, and employing advanced tool materials with enhanced mechanical properties are being explored. Additionally, numerical modeling and simulation techniques, including finite element analysis, offer valuable insights into stress distribution and failure mechanisms, enabling more targeted design improvements. In summary, the challenge of extending punching tool lifespan in industrial settings is closely linked to the reduction in maximum punching forces and the mitigation of damage mechanisms such as chipping, cracking, and swelling. Understanding the interplay between tool geometry, material properties, and operational forces is essential for developing effective solutions that support the demands of high-volume, cost-efficient manufacturing. For this purpose, based on previous collaborative efforts [2] and within a new partnership initiative possessing subject-specific infrastructure and expertise, numerical simulations were carried out at the Sfax University in Tunisia using advanced finite element methods and experimental research and material testing were conducted at Kocaeli University in Türkiye and Silesian University of Technology in Poland. This collaboration merged strong numerical modeling skills with metallurgical and experimental capabilities, enabling comprehensive validation and optimization of punch shaft designs to reduce punching force and enhance tool life.

### 2.2. Numerical Model

The stress–strain behavior of metallic materials under complex loading conditions can be effectively described using the Johnson-Cook constitutive model. This model is particularly well-suited for simulating material response under conditions involving large plastic deformations, elevated temperatures, and high strain rates, which are common in many manufacturing and impact processes [39]. A critical aspect of the Johnson-Cook framework is its incorporation of stress triaxiality as a damage criterion, enabling the prediction of fracture initiation and propagation in ductile materials. Johnson and Cook established that the fracture strain, which defines the onset of material failure, is highly dependent on three principal factors: the stress triaxiality ratio [40], the strain rate [41], and the temperature [42]. These dependencies are captured through a relatively simple yet robust empirical model that combines three multiplicative terms to represent the flow behavior of metals. The flow stress model is expressed as Equation (1) [43]:

(1) $$\sigma =(A+B\varepsilon^{n})\left(1+C\ln\frac{\dot{\varepsilon }}{{\dot{\varepsilon }}_{r}}\right)\left(1-\frac{T-T_{r}}{T_{m}-T_{r}}\right)$$

In this equation, *σ* denotes the equivalent flow stress, while *ε* represents the equivalent plastic strain. The material constants *A*, *B*, *n*, *C*, and *m* correspond to the yield stress under reference conditions, strain hardening constant, strain hardening exponent, strain rate sensitivity coefficient, and thermal softening exponent, respectively. The parameters *T_m_* and *T* indicate the melting temperature of the material and the current deformation temperature, respectively. The terms $\dot{\varepsilon }$_r_ and *T_r_* refer to the reference strain rate and reference temperature, which are typically set to 1.0 s^−1^ and 1298 K, respectively, in this study. In Equation (1) [44], the three terms—from left to right—serve the following distinct roles:

- First term: Characterizes the elastoplastic behavior according to Ludwik’s law, capturing the strain hardening effect.
- Second term: Accounts for viscoplastic behavior, specifically the strengthening effect due to strain rate sensitivity.
- Third term: Quantifies the influence of temperature, reflecting the material’s thermal softening behavior during deformation.

To predict material failure, a Johnson-Cook fracture or damage model is adopted, which describes the equivalent plastic strain at damage onset, ε_f_, as a function of stress triaxiality, strain rate, and temperature. The fracture model is mathematically represented as Equation (2) [45]:

(2) $$\varepsilon_{f}=\left[D_{1}+D_{2}exp\left(D_{3}\left(\frac{\overset{`}{\varepsilon }}{\overset{`}{\varepsilon_{r}}}\right)\right)\right]\left[1+D_{4}ln\frac{\overset{`}{\varepsilon }}{\overset{`}{\varepsilon_{r}}}\right]\left[1+D_{5}\frac{T-T_{r}}{T_{m}-T_{r}}\right]$$

where *D*_1_ through *D*_5_ are the damage model constants: *D*_1_ represents the initial failure strain, *D*_2_ is an exponential factor, *D*_3_ relates to the influence of stress triaxiality, *D*_4_ characterizes the strain rate sensitivity of damage, and *D*_5_ accounts for the temperature dependence of failure. The variables σ_m_ and σ_eq_ denote the mean (hydrostatic) stress and the equivalent (von Mises) stress, respectively. Table 2 compiles the Johnson-Cook constitutive and damage parameters for AISI D2 and S500MC steels, sourced from the literature [46,47,48,49,50]. These parameters are fundamental inputs for finite element simulations that aim to replicate the mechanical response and failure behavior of these materials under industrial forming conditions.

The numerical simulation conducted in this study is grounded on the Johnson-Cook constitutive and damage parameters specifically calibrated for AISI D2 and S500MC steels. These parameter sets enable an accurate representation of the material behavior under the complex loading conditions inherent in the punching process. Punching operations are characterized by the generation of extremely intense plastic deformations localized within very small zones of the workpiece, particularly within the shear zone [51]. This zone experiences not only high strain rates but also significant temperature elevations due to adiabatic heating caused by rapid deformation and frictional effects [52]. Given the severity and localized nature of these deformation conditions, a robust and precise computational approach is required to capture the transient mechanical and thermal responses of both the punch and the sheet material. Accordingly, an explicit dynamic finite element simulation approach has been selected for modeling the punching process. Explicit simulation methods are especially well-suited for problems involving high strain rates, severe localized deformations, and complex contact interactions, as they compute the solution incrementally through explicit time integration schemes that can efficiently handle nonlinearities and rapid transient events. The explicit simulation framework allows for detailed tracking of the evolution of stress, strain, temperature, and damage within the punch and the workpiece throughout the punching cycle [53]. This approach facilitates the identification of critical regions susceptible to failure and wear, as well as the quantification of process parameters’ effects on punching force and tool life [54]. By leveraging the Johnson-Cook model within this explicit simulation, the interplay between strain hardening, strain rate sensitivity, and thermal softening is dynamically accounted for, providing a realistic and comprehensive understanding of the material and tool behavior under operational conditions.

The choice of an explicit simulation methodology, coupled with material models based on Johnson-Cook parameters for AISI D2 and S500MC steels, ensures a high-fidelity representation of the punching process. This enables the investigation of the complex mechanical and thermal phenomena governing tool wear, deformation behavior, and failure mechanisms in high-strain-rate and high-temperature environments characteristic of industrial punching operations.

### 2.3. Designed Punching Tools

**Figure 2:**
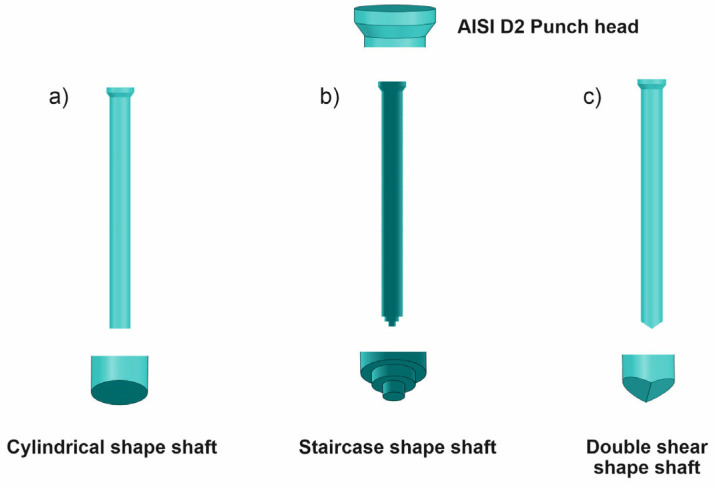
Punching tool shapes namely (**a**) blank punch with cylindrical shaft (**b**) First solution with staircase shaft and (**c**) Second solution with double shear angle.

Figure 2 illustrates the original blank punch alongside two newly proposed design modifications aimed at reducing the punching force and consequently extending the lifespan of punching tools. These design improvements primarily focus on the geometry of the punch shaft, which plays a crucial role in the stress distribution and deformation mechanisms during the punching process. The first modified design retains the overall geometry of the original blank punch but introduces a staircase-shaped shaft. This shape is characterized by an initial reduced diameter at the punch tip where it contacts the upper surface of the sheet, followed by a subsequent larger diameter step that returns the shaft to its original 3 mm diameter. This stepped reduction in diameter is intended to facilitate a gradual penetration of the punch into the S500MC steel sheet, thereby mitigating the risk of sudden stress concentrations and chipping at the punch edge. By controlling the deformation progression in discrete stages, this design aims to reduce the instantaneous punching force and distribute the load more evenly along the shaft, which can help in minimizing localized tool wear and damage.

The second proposed design introduces a double shear geometry on the punch shaft, which effectively creates a V-shaped profile at the upper section of the sheet during punching. This double shear configuration allows the punch to engage the material in a more controlled manner, reducing the abrupt increase in deformation forces commonly experienced with traditional cylindrical punches. The V-shaped shear action helps to absorb and dissipate the mechanical shocks induced during punching, thereby lowering the peak punching force and enhancing the tool’s resistance to fatigue and failure. Importantly, this design maintains symmetry in the punch geometry, preserving the mechanical balance of the AISI D2 tool, which is critical for stable operation and uniform load distribution. Both design modifications aim to address a key industrial challenge: the frequent need to sharpen punching tools due to wear and damage, which interrupts production and significantly increases manufacturing costs. These interruptions are particularly detrimental in large series production where downtime directly impacts throughput and profitability. By altering the punch shaft geometry to enable a staged and less force-intensive punching process, these designs reduce the severity of cold work deformation on the tool, thereby extending its usable life and minimizing the frequency of maintenance. Furthermore, the staged punching approach enabled by these designs facilitates the formation of high-quality holes with diameters of 3 mm. The incremental deformation helps prevent the coalescence of cracks and other defects that typically arise during high-force punching, resulting in cleaner edges and improved dimensional accuracy. This improvement in hole quality is particularly important in applications requiring tight tolerance and consistent performance. The staircase-shaped and double shear punch designs represent promising solutions for enhancing tool life and process efficiency in cold punching operations. By moderating the punching force through geometric modifications, these designs not only reduce tool wear and production downtime but also contribute to improved product quality and cost-effectiveness in industrial manufacturing settings.

### 2.4. RSM Method

Determining the impact of punching parameters on the maximum force required during cold forming is of paramount importance, as it directly influences the lifespan and durability of punching tools. Among the various process parameters, punching speed plays a critical role due to its strong correlation with tool wear mechanisms and fatigue life [55]. By understanding how variations in punch velocity affect the mechanical interaction between the tool and the workpiece, manufacturers can optimize process conditions to extend tool life while maintaining product quality and dimensional accuracy [56].

In this study, the investigation is deliberately focused on punching speed, recognizing it as a key factor that governs both the dynamic response of the punch and the resulting material deformation behavior. The finished product’s quality, manifested through surface finish, dimensional precision, and absence of defects, is intrinsically linked to the punch’s velocity profile during operation. Variations in punching speed can alter strain rates, temperature development, and stress distributions within the material and tooling, all of which affect tool wear and hole quality. To rigorously explore the relationship between punching speed and maximum punching force, this work employs Response Surface Methodology (RSM), a statistical and mathematical technique designed to model and analyze the influence of one or more explanatory variables (factors) on one or more response variables. RSM offers a practical advantage by providing an empirical model that approximates complex relationships through designed experiments, enabling efficient exploration of the parameter space without exhaustive trial-and-error testing. The adoption of RSM is motivated by its efficiency and reliability compared to alternative methods, which may be cumbersome, time-consuming, or less robust in capturing nonlinear interactions among variables. In the context of punching parameter optimization, RSM has emerged as a widely accepted tool due to its ability to generate predictive models, optimize conditions, and visualize response surfaces that reveal insights into the underlying process mechanics [57]. Beyond statistical modeling, this study integrates RSM with finite element analysis to monitor the progression of the punching tool as it penetrates the material. Particular attention is given to the evolution of the Von Mises stress distribution, a crucial indicator of yielding and plastic deformation within the workpiece and tool. The investigation tracks the development and propagation of stress concentration zones along the punch axis, which are critical factors influencing tool wear, potential failure, and hole formation quality. By coupling experimental design and numerical simulation, this approach aims to elucidate the complex interactions between punching speed, material deformation, and stress evolution. The goal is to identify punching velocities that minimize maximum punching force and stress concentrations, thereby enhancing tool lifespan while ensuring high-quality, dimensionally accurate product output.

### 2.5. Von Mises Distribution in AISI D2 Punch

#### 2.5.1. Blank Shaft-Shape Punch

**Figure 3:**
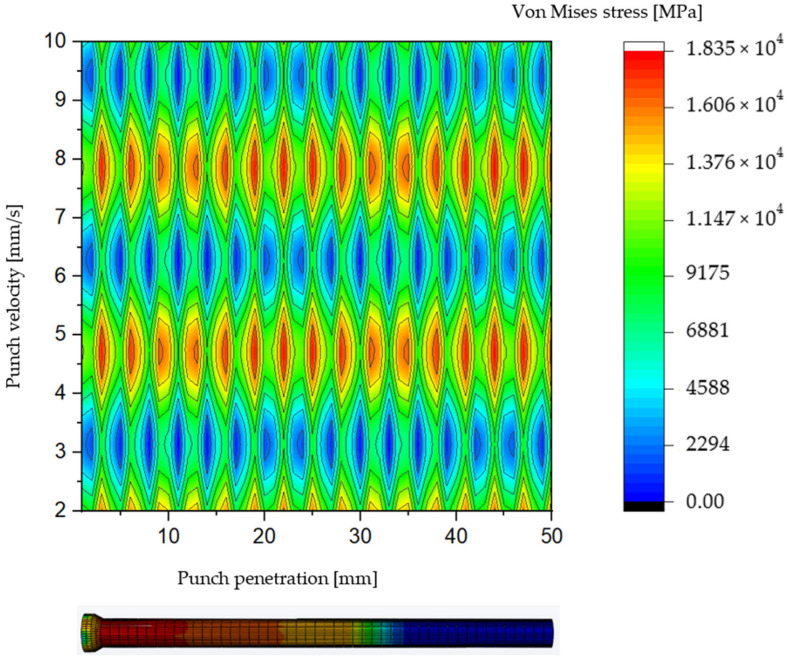
Color filled contour plot for blank punching tool.

The blank punch utilized in this study features a cylindrical shaft with a diameter of 3 mm. This portion, referred to as the active part of the punch, serves as the primary component responsible for penetrating and punching through the S500MC sheet metal. The investigation into the impact of punching velocity on the punching force during the tool’s penetration into the material is conducted as a control case, providing a baseline for comparison against the alternative punch designs introduced in this work. As illustrated in Figure 3, the evolution of Von Mises stress is analyzed as a function of both punch penetration depth and punching velocity. This representation effectively captures the influence of punching velocity on the distribution and magnitude of stress experienced during the punching operation. The Von Mises stress criterion is particularly relevant in this context as it quantifies the equivalent stress responsible for yielding under complex loading conditions, offering insight into the plastic deformation behavior of the tool and workpiece. The stress analysis reveals distinct patterns correlated with different punching velocities. Specifically, regions where Von Mises stress is reduced, depicted in blue, are predominantly observed at punching velocities of 3, 6, and 9 mm/s within penetration intervals of [10 mm, 20 mm] and [30 mm, 40 mm]. These zones of reduced stress suggest temporary relief in mechanical loading, which may contribute to decreased tool wear when operating within these velocity ranges. Conversely, punching velocities of 4, 7, and 10 mm/s exhibit pronounced peaks in Von Mises stress, indicating elevated levels of mechanical constraint on the punching tool. These stress concentrations generate significant compressive stresses, particularly notable at punch penetration depths of approximately 10 mm and 45 mm. The presence of such stress peaks suggests critical loading conditions that may accelerate tool fatigue and wear, potentially leading to premature failure. Overall, the distribution of Von Mises stress along the punch shaft during penetration is observed to be neither spatially homogeneous nor symmetrically balanced. This non-uniformity in stress distribution highlights the complex interaction between punching velocity, tool geometry, and material deformation. Understanding these stress patterns is essential for optimizing punching parameters to reduce maximum punching forces, improve tool life, and ensure consistent hole quality.

**Figure 4:**
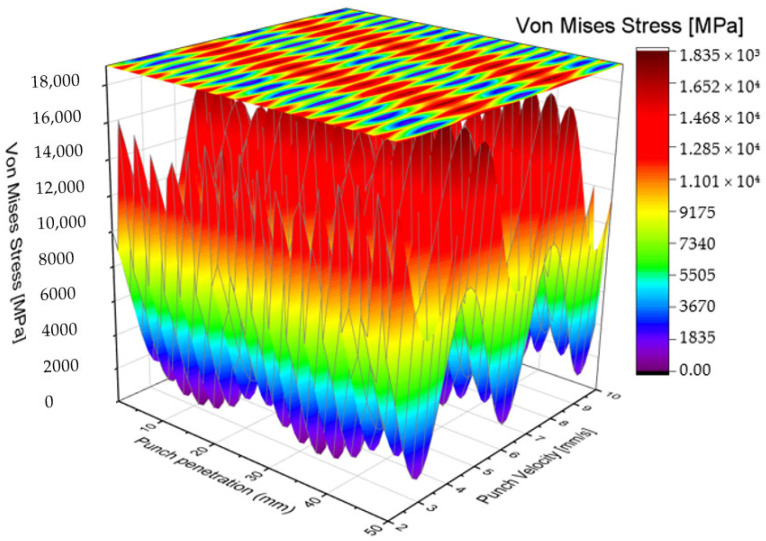
Vons Mises stress distribution for AISI D2 blank punch depending on tool penetration and velocity.

The zones of stress concentration exhibit notable variation across different punching speeds, yet they are predominantly localized on the shaft and head regions of the punching tool. These highly stressed areas are critical, as they are the primary sites where mechanical failure mechanisms such as swelling, chipping, and fatigue cracking tend to initiate. Consequently, particular emphasis is placed on optimizing the design of the punch shaft to achieve a more uniform and balanced stress distribution, thereby mitigating the risk of localized damage and enhancing the overall durability of the tool. As depicted in Figure 4, regions highlighted in red correspond to areas where the Von Mises stress reaches values as high as 1835 MPa. Such elevated stress levels exceed the typical yield and fatigue limits of the punching tool material, rendering the tool incapable of withstanding the imposed mechanical loads during the punching operation. This excessive stress concentration is a primary factor contributing to premature tool damage, which in turn can cause unexpected production stoppages and significant economic losses due to downtime and tooling replacement costs. Addressing this critical challenge, the present work employs advanced design methodologies utilizing 3D computer-aided design (CAD) software coupled with FEM. This integrated approach allows for systematic exploration and optimization of punch geometry with the objective of redistributing stress more evenly along the tool shaft and head. By simulating various design iterations under realistic punching conditions, the study seeks to identify geometrical modifications that reduce peak stresses and alleviate critical concentrations responsible for tool failure. Through this design-driven computational investigation, improvements in punch geometry can be validated prior to physical prototyping, thereby reducing development time and cost. The goal is to develop punch designs that maintain structural integrity under high punching forces, minimize wear and damage, and support sustained high-volume production without interruptions.

#### 2.5.2. Staircase Shaft-Shape Punch

Numerous solutions have been proposed by researchers in the field of cold forming aimed at optimizing punching processes; however, the results have often been inconsistent or unsatisfactory in certain applications [58]. This variability arises largely from the fact that each geometric shape or feature to be formed requires a unique set of processing parameters. Even minor adjustments to one parameter can have significant and complex effects on other process variables, leading to challenges in achieving universally optimal conditions. Consequently, there remains a pressing need for innovative punch designs, particularly focused on the active part of the punch where material interaction is most critical. In response to these challenges, the present work introduces novel punch geometries, with particular emphasis on a staircase-shaped shaft design, analyzed through comprehensive FEM simulations. This design features two stepped diameters along the punch shaft, enabling staged penetration into the material. Initially, a smaller diameter creates a preliminary hole, which serves to significantly reduce the punching force required. Subsequently, a larger diameter enlarges the hole incrementally until the final 3 mm diameter is fully formed. This gradual material displacement approach aims to distribute the deformation more evenly and reduce peak stresses on the punch.

**Figure 5:**
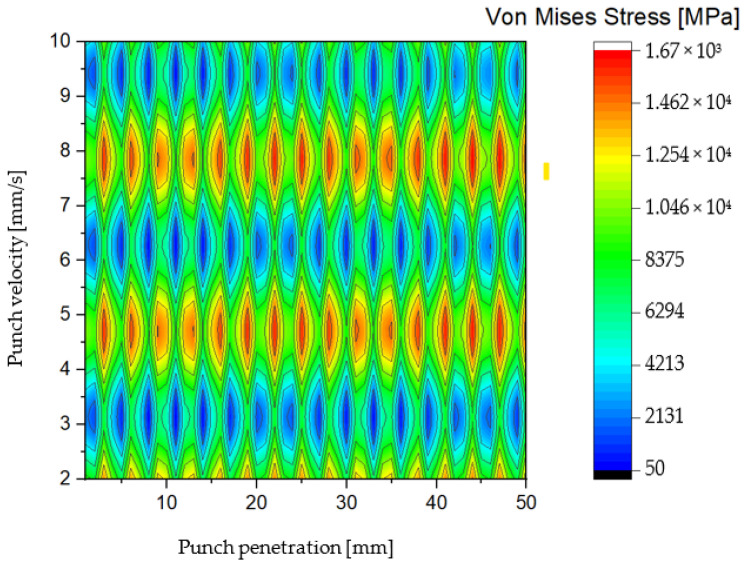
Color filled contour plot for staircase shaft shaped punch.

**Figure 6:**
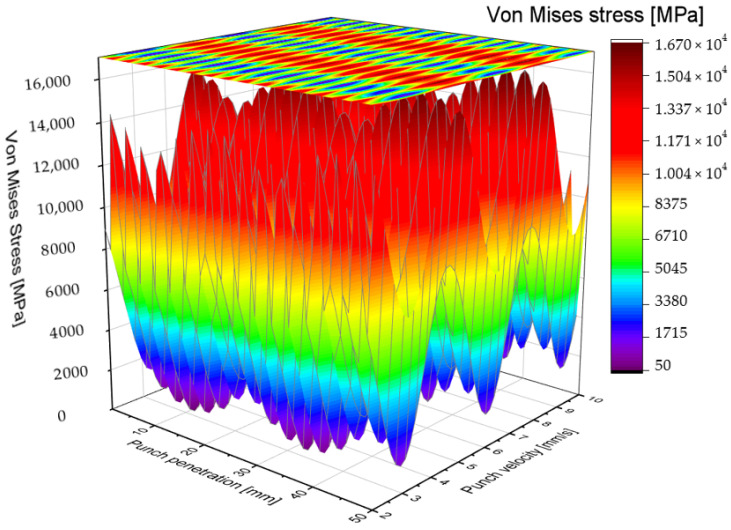
Vons Mises stress distribution for AISI D2 blank punch depending on Tool penetration and velocity.

Figure 5 and Figure 6 illustrate the distribution of Von Mises stress as a function of punching speed and punch penetration depth for the staircase-shaped punch. The stress maps reveal trends like those observed with the traditional blank punch; however, a notable reduction in Von Mises stress magnitude is evident across the examined range of punching speeds. Specifically, the maximum Von Mises stress recorded for the modified punch does not exceed approximately 1670 MPa, representing a significant reduction of nearly 2000 MPa compared to the peak stresses observed in the blank punch configuration. Moreover, the staircase-shaped punch exhibits minimum stress concentrations particularly at punching speeds of 3, 6, and 9 mm/s, which aligns with regions where the blank punch previously showed stress reduction zones. These findings suggest that the stepped shaft design effectively mitigates critical stress concentrations, thereby lowering the mechanical load on the tool and potentially extending its operational lifespan.

When considering the punch length as a reference parameter, the minimum Von Mises stress for the staircase-shaped punch is observed within the intervals of [20 mm, 30 mm] and [40 mm, 50 mm]. This finding represents a notable deviation from the behavior exhibited by the blank punch, where stress minimums were distributed differently along the shaft. The presence of these distinct low-stress zones indicates that the modified punch geometry fundamentally alters the stress distribution pattern during material penetration. One of the salient advantages of this design is its inherent geometric symmetry, particularly when viewed from the punch tip or end. This symmetry contributes significantly to mechanical balance during the punching operation, ensuring that the tool experiences uniform loading and minimizing the risk of uneven wear or bending moments. Such balanced force distribution is critical in maintaining the structural integrity of the punch and achieving consistent hole quality in the S500MC sheet metal. Moreover, the staged hole-forming mechanism inherent in the staircase-shaped shaft design offers an additional benefit: it reduces the risk of chipping at the punch tip.

By progressively enlarging the hole through incremental diameter steps, the punch avoids abrupt force spikes and localized overloading that commonly lead to edge damage in conventional punches. This controlled penetration mechanism helps preserve the sharpness and dimensional accuracy of the punch’s active surface over extended usage. The distribution of Von Mises stresses along the punch shaft in this design is notably quasi-homogeneous, which effectively prevents the development of critical stress concentrations that are often precursors to crack initiation and tool failure. The reduced presence of stress risers not only improves the mechanical resilience of the punch but also contributes to more stable and predictable tool performance during repetitive punching cycles. In addition to the improved stress distribution, the substantial reduction in maximum punching stress afforded by this design is a critical factor in enhancing tool lifespan. Lower peak stresses translate directly into diminished fatigue damage and slower wear rates, thereby extending the service life of the punching tool. This advantage supports higher production throughput and reduces maintenance costs, as corroborated by earlier studies on tool durability [59].

#### 2.5.3. Double Shaft-Shape Punch

**Figure 7:**
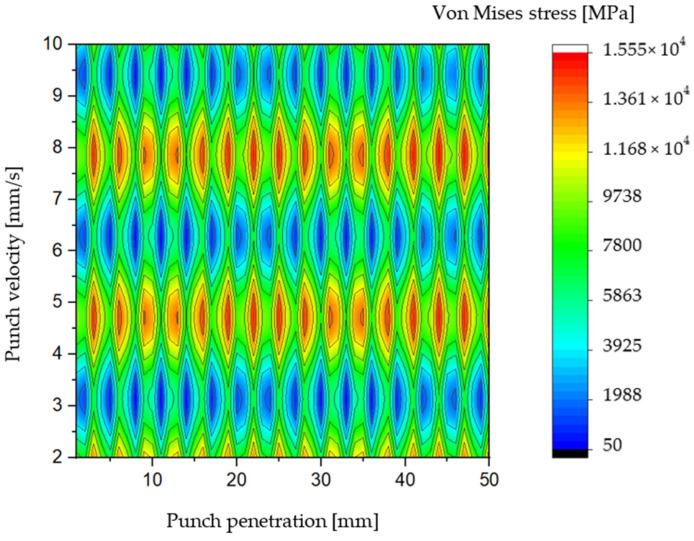
Color filled contour plot for double-shear shaped punch.

**Figure 8:**
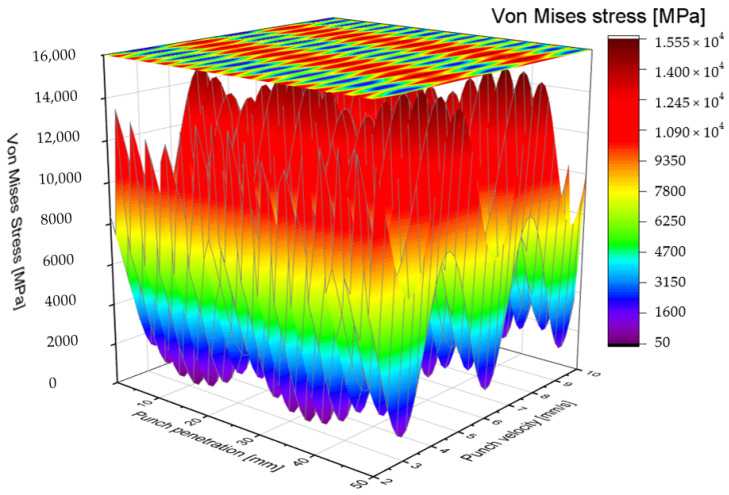
Vons Mises stress distribution for AISI D2 blank punch depending on tool penetration and velocity.

The second solution proposed in this study involves a punch shaft featuring a double-shear angle design, characterized by a distinctive V-shaped geometry. This configuration facilitates a gradual punching process by smoothing the impact forces during the initial contact between the punch and the S500MC sheet metal. The V-shaped profile effectively mitigates sudden shocks, allowing for a more controlled material deformation and progressive hole formation, which are critical for reducing tool wear and improving hole quality [60]. Figure 7 and Figure 8 illustrate the Von Mises stress distribution as a function of both punching speed and punch penetration depth for the double-shear angle punch. The stress maps reveal a substantial reduction in the peak Von Mises stress compared to the traditional blank punch. Specifically, the maximum Von Mises stress for the double-shear design does not exceed approximately 1555 MPa, a notable decrease of around 400 MPa relative to the blank punch configuration. This reduction signifies a meaningful alleviation of mechanical loads experienced by the punch during operation. Examining the stress distribution along the length of the AISI D2 punch reveals that the Von Mises stress reaches its minimum values primarily within the penetration intervals of [0 mm, 10 mm] and [40 mm, 50 mm]. Notably, the stress magnitude decreases considerably at punching speeds of 3, 6, and 9.5 mm/s, with a near-zero Von Mises stress observed at a speed close to 10 mm/s and a punch length of approximately 45 mm. This finding suggests that the double-shear angle punch exhibits enhanced mechanical stability under specific operational conditions, particularly at higher punching speeds and near full penetration. Furthermore, the overall distribution of Von Mises stress along the punch shaft demonstrates improved homogeneity and stability compared to other punch designs. This more uniform stress profile reduces localized stress concentrations, thereby lowering the risk of premature tool failure due to fatigue or chipping. The stability of stress distribution also implies that the double-shear angle design can maintain consistent performance over extended production cycles. Through detailed numerical simulations based on FEM, this study confirms that the double-shear angle punch represents an optimal solution when considering multiple performance criteria. It achieves a mechanically balanced punching action, facilitates a controlled and gradual progression of material deformation, and significantly reduces the maximum equivalent stress experienced by the tool. Collectively, these attributes contribute to enhanced tool longevity, improved hole quality, and increased process reliability.

During the punching process using the double-shear angle punch design, the tool initiates contact by creating a localized indentation or trace on the upper surface of the S500MC sheet within the elastic deformation zone. As the process progresses, the material beneath the punch transitions into the plastic deformation zone, where the slug is gradually detached from the sheet. The procedure concludes with the formation of a precise 3 mm diameter hole. These distinct punching stages correspond closely with the observed distribution of Von Mises stress, allowing for an insightful analogy between the mechanical stress evolution and the physical deformation sequence. Detailed analysis reveals that the AISI D2 punch experiences particularly intense compressive stresses at punching speeds of approximately 3.6 mm/s and 9 mm/s. These elevated stress levels are critical because they increase the likelihood of accelerated tool wear and mechanical failure. Consequently, it is advisable to avoid operating within these specific velocity ranges to minimize the punching force exerted on the tool, thereby enhancing its lifespan and maintaining operational reliability. Premature punch damage, characterized by phenomena such as chipping, cracking, and plastic deformation, can be triggered by abrupt increases in cold forming forces. The underlying mechanism of this failure mode has been comprehensively described in prior studies [61], which highlight the sensitivity of tool integrity to sudden load spikes during punching operations. These force surges generate stress concentrations that exceed the material’s endurance limit, leading to early onset of fatigue and catastrophic tool failure. Given these considerations, the selection of an optimal punching solution is contingent upon its ability to effectively reduce the maximum punching force and mitigate sudden force fluctuations. Among the designs investigated, the double-shear angle punch demonstrates superior capacity to moderate force progression and maintain stress levels within safe thresholds. Therefore, this solution is identified as the most favorable for industrial application, offering the potential to extend punch service life, reduce downtime, and improve overall process efficiency.

## 3. Results and Discussion

**Figure 9:**
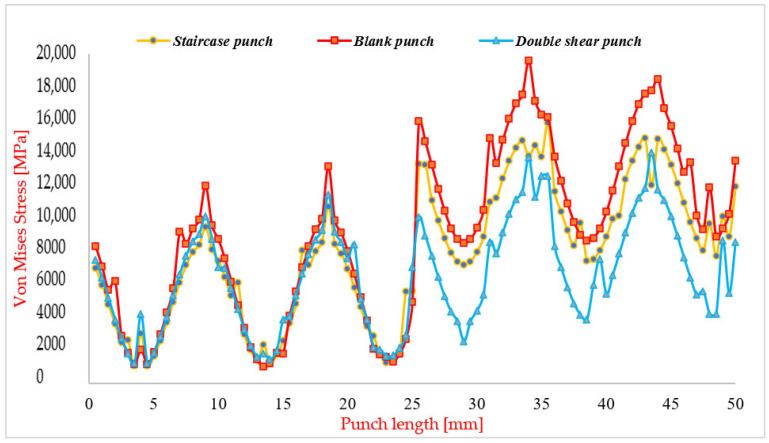
Von Mises stress distribution for three punches, namely blank punch; Staircase punch; Double shear punch [62].

FEM employed in this study proves to be an exceptionally effective computational tool for systematically comparing the performance of the proposed punch designs without the necessity of time-consuming and costly physical experiments. By leveraging FEM, it becomes possible to accurately predict and quantify critical mechanical parameters, such as maximum and minimum forces, stress distributions, and notably the Von Mises equivalent stress, which are essential indicators of tool performance and durability under operational conditions. Figure 9 illustrates the evolution of Von Mises stress for the two newly proposed punch geometries, the staircase-shaped shaft and the double-shear angle shaft, across the punching process. This comparative visualization highlights the distinct stress behaviors associated with each design, providing valuable insight into their relative effectiveness in mitigating stress concentrations and reducing peak loads on the punching tool. Through these simulations, FEM enables a detailed assessment of how each design influences the mechanical environment experienced by the punch during sheet metal deformation. This insight facilitates informed decision-making regarding optimal punch geometry, guiding the selection of solutions that enhance tool lifespan, improve manufacturing efficiency, and maintain product quality.

### 3.1. Numerical Results

The FEM utilized in this study seems to be a good method for comparing the performance of the proposed punch designs, as it reduces the need for extensive and time-consuming physical experimentation. FEM facilitates the precise determination of critical mechanical parameters, including the maximum and minimum stress values, with particular emphasis on the Von Mises equivalent stress, a key indicator of yielding and potential failure in materials. Figure 9 presents the distribution of Von Mises stress for the newly proposed punch designs as a function of punch length and punching speed. This visualization offers valuable insights into the stress behavior throughout the punching process, enabling a direct comparison of how each design influences tool loading conditions. Previous investigations into the optimization of punching parameters have been extensive; however, their findings often vary widely and can sometimes be contradictory due to differences in experimental setups, material properties, and process conditions. In contrast, the present work focuses specifically on the evolution of Von Mises stress as a reliable metric for evaluating the effectiveness of punch designs in reducing mechanical stresses. Analysis of the Von Mises stress evolution curve for the blank punch (i.e., the traditional cylindrical punch without modifications) reveals a bipartite behavior. For punch lengths between 0 and 25 mm, corresponding to the punch head region, the Von Mises stress remains relatively low and does not exceed approximately 14,000 MPa. Beyond this region, from 25 mm to 50 mm along the punch shaft, the stress rises sharply, reaching values up to 19,000 MPa. This dramatic increase indicates that the punch shaft is subjected to intense compressive stress, a finding corroborated by previous studies [63]. Such high stress concentrations on the shaft highlight the critical need for targeted design modifications focused specifically on this region to mitigate stress and improve tool durability. The staircase-shaped punch demonstrates a stress distribution curve similar in shape to the blank punch but with a significant reduction of approximately 3000 MPa in Von Mises stress. This reduction suggests that the staircase design effectively lowers the mechanical load on the punch, facilitating the punching of a 3 mm diameter hole with reduced risk of tool damage. Consequently, the expected extension in punch lifespan could lead to substantial cost savings in production by minimizing tool replacement and maintenance. The double-shear angle design exhibits an even more favorable stress profile, characterized by symmetry and reduced contact area with the sheet metal upper surface. This geometry contributes to a pronounced decrease in Von Mises stress by more than 4500 MPa compared to the blank punch. The combination of lower peak stresses and balanced stress distribution positions the double-shear punch as the optimal solution among those studied. Its adoption is anticipated to significantly reduce the likelihood of premature punch failure, thereby enhancing reliability and efficiency in cold punching operations.

### 3.2. Experimental Validation

To conduct the experimental tests, a tensile testing machine with a maximum capacity of 50 kN was employed. While this machine is primarily designed for compression testing, it can replicate the movement of a hydraulic press. At the company facility, the process is not automated; instead, an operator manually inserts the sheet metal and initiates each cycle. The punching operation concludes automatically once the predefined stroke of the mobile part is reached.

**Figure 10:**
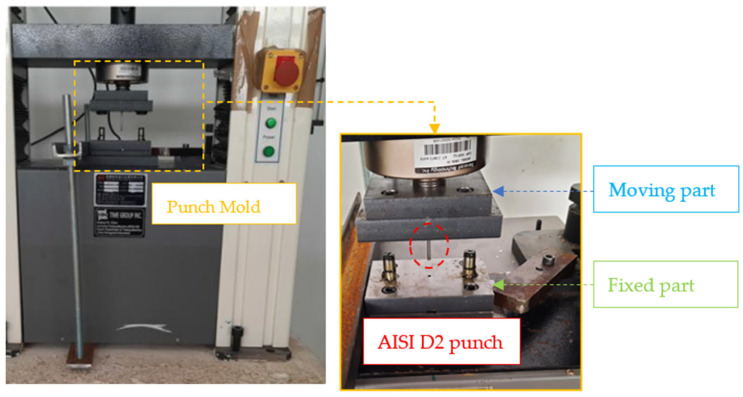
Prototype punching mold mounted on the traction machine.

A similar procedure is followed in the laboratory setting. The cycle begins by pressing the “down” button, and the descent of the mobile part of the tensile machine is halted as soon as the punching sound is heard, indicating the completion of the punching process. Using a milling machine, the sheet metal thickness was reduced from 6 mm to 3 mm to maintain the same punch-diameter-to-sheet-thickness ratio, as depicted in Figure 10.

The mobile part of the mold was attached to the tensile machine based on the machine’s existing fastening elements. The mobile part of the tensile machine features an M22 threaded rod with a length of 40 mm.

The stationary part of the mold was securely fixed to the tensile machine using a clamp. This method was necessary due to the presence of a threaded hole on the fixed part of the machine. To ensure proper alignment of the lower base plate, a centering pin was used to eliminate two translational and two rotational degrees of freedom, with their axes located on the fixed part of tensile machine. The remaining degrees of freedom were constrained by the clamp. However, during mold opening, the lower base plate tended to lift with the mobile part. This issue was resolved by using two threaded rods and two steel strips, each containing two threaded holes. This solution effectively secured the end of the lower base plate, as the opposite side remained fixed by the clamp. Through these measures, the mold was successfully mounted onto the tensile machine.

During each test cycle, the machine was activated to close the mold, completing the punching process once a fully formed hole was achieved. Following each cycle, the mold was reopened, and the sheet strip was manually advanced by 1 cm. This step was taken to mitigate the edge effect and ensure consistent testing conditions for subsequent cycles.

To minimize friction, the guide elements of the mold were lubricated with grease every 20 cycles. Additionally, the active part of the punch was treated with engine oil to facilitate smoother movement. To further optimize the process, the highest available punching velocity on the tensile machine was utilized, with the aim of reducing the required punching force.

**Figure 11:**
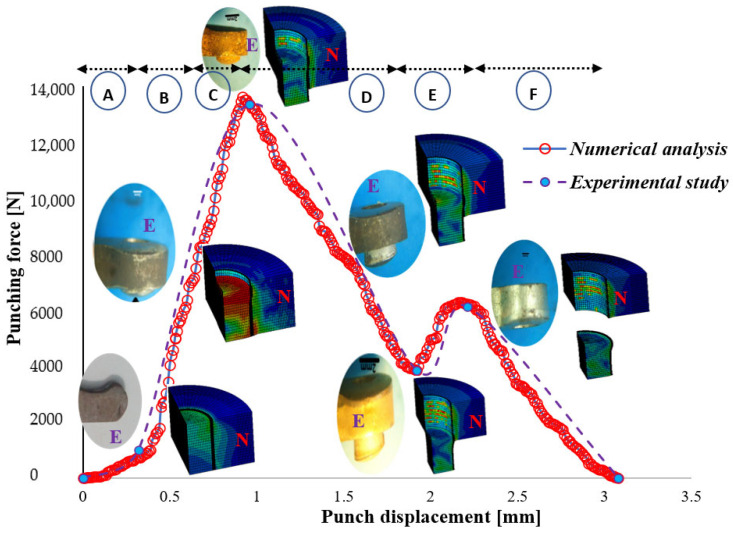
Comparison of experimental (E) and numerical (N) punching force versus punch displacement curves at a punch velocity of 10 mm/s and a punch-die clearance of 0.3 mm, namely (A) Initial Contact; (B) Elastic Deformation; (C) Plastic Deformation and Maximum Punching Force; (D) Material Damage Initiation; (E) Slug Detachment and Frictional Effects and (F) Slug Ejection and Completion [62].

The evolution of the punching force as a function of punch displacement under dry conditions, depicted in Figure 11, can be distinctly segmented into six sequential phases labeled A through F. These phases collectively characterize the complex mechanical interactions and material responses during the punching process, encompassing punch-shaft contact, elastic and plastic deformation stages, material damage onset, slug extraction, and frictional effects between the punch, slug, and die.

***Phase A: Initial Contact***

In the initial phase, the punch shaft makes first contact with the upper surface of the metrix head key S500MC sheet. During this stage, the press’s movable component completes its stroke, causing the punch to lightly touch the sheet without penetrating the material. The punching force increases gradually and slightly because of the reaction force exerted by the sheet metal on the punch. This initial contact is critical for preventing lateral sliding and ensuring that the punch remains properly balanced and centered on the sheet. The stability achieved during this phase significantly influences the accuracy and quality of the final hole [64].

***Phase B: Elastic Deformation***

Following the initial contact, the sheet metal undergoes elastic deformation. In this phase, material deformation is reversible, allowing the sheet to return to its original shape if the load is removed. The punching force rises progressively as the punch slowly penetrates the material without causing permanent deformation or detachment of any metal particles. The contact between the punch and the sheet’s upper surface is maintained, and the elastic properties of the metrix head key sheet play a vital role in guiding the punch and sustaining the cold forming operation. The support provided by the elastic deformation zone ensures controlled progression of the punch.

***Phase C: Plastic Deformation and Maximum Punching Force***

Upon further penetration, the sheet metal enters the plastic deformation regime, characteristic of ductile materials undergoing permanent deformation. This phase corresponds to the peak punching force, commonly referred to as the shearing force, which signifies the onset of material separation and acceleration of metal particles (slug formation). The punch experiences intense compressive stresses as it is pressed between the XC 90 back-up plate and the S500 MC sheet. The maximum punching force recorded is approximately 13,620 N at a punch displacement of 0.9 mm, coinciding with the full formation of the die and punch shoulder radii within the sheet. At this point, the sheet exerts substantial reaction forces on the punch’s lower region, inducing severe mechanical loading and initiating plastic flow, evidenced by bulging in the die area [65]. This deformation highlights the sheet’s plastic response to the applied punch force.

***Phase D: Material Damage Initiation***

Following the peak force, the punching force begins to decline gradually, marking the initiation of material damage. Micro-cracks form on the upper surface of the sheet, and scratches develop at the interface between the punch and sheet edges. A sudden reduction in force at nearly constant punch displacement is observed, attributable to localized thermal effects such as burning near the cutting edges, punch, and die sides. The punching force is sufficient to overcome the material’s mechanical resistance, initiating the breakage of atomic bonds within the sheet. This phase signals the propagation of cracks and the onset of material failure, which facilitates slug detachment.

***Phase E: Slug Detachment and Frictional Effects***

This phase encapsulates the complexity inherent in the punching process, involving multiple interacting parameters. Notably, the stripping phase, where the slug is removed from the punch, is not considered in the current simulation or experimental setup, as the die matrix was designed without draft angles to simplify the numerical model and avoid precision constraints. As illustrated, the punching force decreases from the previous damage phase to approximately 4000 N, indicating the successful detachment of the slug from the sheet. However, the slug continues to rub against the inner die surface, causing an increase in force up to 6000 N to overcome die-slug friction. The effective slug thickness is less than the nominal 3 mm due to compressive stress, explaining the force peak observed at a punch displacement of 2.2 mm.

***Phase F: Slug Ejection and Completion***

The final phase is marked by a sudden drop of the punching force to zero, signaling the ejection of the slug from the die cavity and the completion of the punching operation. At this stage, a 6 mm diameter hole is fully formed in the metrix head key sheet. The punch shaft continues to move through the sheet and into the die, with the force dropping after penetrating the 3 mm sheet thickness. The residual force corresponds to the effort required to fully extract the slug from the die cavity, concluding the punching cycle.

**Figure 12:**
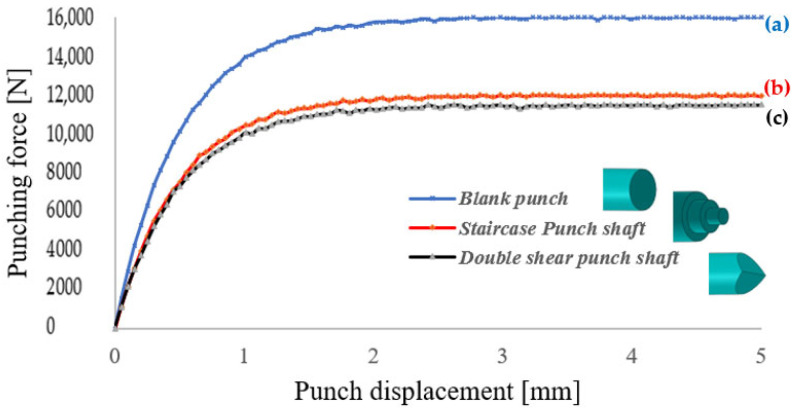
Comparison of punching force vs. displacement for blank punch (a), staircase punch (b) and double shear punch (c).

Figure 12 illustrates the relationship between punching force and punch displacement for three distinct punch designs: the blank punch, the staircase punch, and the double shear punch. This comparative analysis provides valuable insight into the mechanical behavior and efficiency of each design under load, highlighting the impact of geometric modifications on force distribution and process stability.

***Blank Punch:*** The blank punch exhibits the highest punching force among the three designs, with a rapid escalation in force culminating in a peak value of approximately 15,000 N before reaching a plateau. This sharp rise indicates a concentration of stress within localized regions of the material and punch interface, reflecting inefficient load distribution. Such force spikes are detrimental, as they increase the likelihood of premature tool wear, chipping, and material failure. The pronounced force peak and abrupt loading characteristics suggest that the blank punch design is less optimized, especially in applications where controlled force application and material integrity are critical considerations.

***Staircase Punch:*** The staircase punch demonstrates a more controlled and gradual force-displacement response relative to the blank punch. The peak force reaches approximately 12,000 N, which is a notable reduction compared to the blank design. The smoother ascent in the force curve reflects a staged load transfer arising from the stepped shaft geometry, which allows the punch to progressively engage the material, mitigating sudden stress concentrations [66]. Although this design improves upon the blank punch by reducing peak force and enhancing load distribution, the relatively high maximum force may still pose challenges in applications demanding minimal force input or where tool longevity is paramount.

***Double Shear Punch:*** The double shear punch emerges as the most mechanically efficient design, achieving the lowest peak punching force of approximately 10,500 N. Its force-displacement curve is characterized by a gradual, steady increase in force with minimal fluctuations, indicative of superior force management and enhanced process stability. This design effectively distributes the punching load over a larger interaction area and stages the deformation process, thereby reducing localized stress concentrations and minimizing dynamic force spikes. The improved force consistency promotes smoother operation, reduces stress-induced tool damage, and enhances material longevity, making the double shear punch particularly well-suited for high-precision and high-volume industrial applications.

***Comparative Analysis:*** All three force-displacement curves originate near zero force at zero displacement, reflecting the initial state before punch contact. However, their trajectories diverge significantly as penetration progresses. The blank punch shows a steep force rise and high peak, the staircase punch exhibits a moderated force increase with a lower peak, and the double shear punch combines the lowest peak force with the most uniform force progression. This comparison underscores the critical influence of punch geometry on operational efficiency and tool performance.

In conclusion, the double shear punch design offers clear advantages over both the blank and staircase designs, primarily through its ability to significantly reduce peak punching force and maintain a predictable, stable force response throughout the punching stroke. These characteristics not only enhance tool life and product quality but also improve process reliability, making the double shear punch the optimal choice for demanding cold forming operations

The selection of punch design—whether stepped, double-shear, or standard cylindrical—significantly influences both machining difficulty and manufacturing costs, which are critical factors in engineering applications.

- **Machining Difficulty:** The **standard cylindrical punch** is the simplest to manufacture due to its uniform geometry, requiring minimal tooling adjustments and straightforward machining processes. In contrast, the **stepped design** introduces additional complexity, as it necessitates precise control over multiple diameters and transitions, increasing the risk of machining errors and requiring advanced tooling. The **double-shear design**, while effective in reducing punching forces, further complicates the process due to its angled shear surfaces, demanding specialized machining techniques and tighter tolerances.
- **Manufacturing Costs:** Costs are directly tied to the complexity of the design. The **standard cylindrical punch** remains the most cost-effective option, as it involves fewer machining steps and lower tool wear. The **stepped punch**, although more expensive due to its multi-stage geometry, can offer cost savings in specific applications where material deformation is more controlled, potentially reducing secondary operations. The **double-shear punch**, while the most expensive to produce due to its intricate geometry, may justify its cost in high-precision applications where force reduction and part quality are paramount.
- **Practical Guidance for Engineering Applications:** For **high-volume production** with standard materials, the cylindrical punch is often the most economical choice. However, in applications requiring **enhanced precision, reduced punching forces, or improved part quality**, the stepped or double-shear designs may be justified despite their higher costs. Engineers should weigh the trade-offs between initial manufacturing costs and long-term benefits, such as reduced tool wear, improved part quality, and lower energy consumption during operation.

This comparative analysis provides a practical framework for selecting the optimal punch design based on specific application requirements, balancing technical performance with economic considerations. Thank you again for highlighting this important aspect.

## 4. Conclusions

In this study, the FEM was employed using ABAQUS software (6.17), complemented by Response Surface Methodology (RSM), to systematically analyze and optimize the punching process. Two innovative punch designs, the staircase shaft and the double-shear angle shaft, were proposed and rigorously evaluated against the conventional blank punch commonly used in industrial settings. The evaluation focused primarily on the distribution of Von Mises stress and the magnitude of punching force, with both numerical simulations and experimental investigations yielding results that demonstrate strong agreement and excellent reproducibility.

The key findings of this investigation can be summarized as follows:

Comparative Performance of Proposed Solutions: Both novel punch designs exhibit similar overall trends in Von Mises stress distribution and punching force behavior, yet distinct differences emerge in the magnitude and location of peak stress regions. These variations are closely linked to operational parameters such as punching speed and punch penetration depth, underscoring the sensitivity of each design to process conditions. Understanding these dependencies is crucial for tailoring punch selection to specific manufacturing scenarios.

Optimality of the Double-Shear Punch: Among the designs assessed, the double-shear punch stands out as the optimal solution. It achieves superior stability in Von Mises stress distribution, significant reductions in peak stress values, and a balanced force profile throughout the hole-forming process. This combination of attributes points to enhanced process efficiency and extended tool life, marking the double-shear punch as a promising candidate for demanding cold punching applications where precision and durability are paramount.

Effectiveness of the Staircase Shaft Design: The staircase shaft punch also demonstrates notable effectiveness in mitigating premature tool damage by reducing stress concentrations that typically initiate wear and failure. This design’s capacity to stage material deformation and distribute loads more evenly renders it particularly suitable for operations prioritizing tool reliability and consistent performance over prolonged production runs.

Overall, the excellent concordance between numerical simulations and experimental data not only validates the fidelity and accuracy of the FEM models but also confirms the reproducibility and robustness of the findings. These comprehensive insights provide a solid foundation for informed decision-making in punch design selection, enabling manufacturers to balance operational efficiency, tool durability, and product quality in alignment with specific industrial requirements.

## Figures and Tables

**Table 1:** Elasticity parameters and density of AISI D2 and S500MC steel.

	E (GPa)	ν	Density (kg/m^3^)
AISI D2	230	0.28	7.9 × 10^3^
S500MC	209	0.28	7.9 × 10^3^

**Table 2:** Johnson-cook model and damage parameters of AISI D2 and S500MC steels.

Parameters	AISI D2	S500MC
A (N/mm^2^)	1490	510
B (N/mm^2^)	660	220
n	0.04	0.28
C	0.29	0.0019
m	0.38	1
$\bar{\dot{\varepsilon 0}}$	1	1
D_1_	0.69103	0.53467
D_2_	0	0
D_3_	0	0
D_4_	−0.03524	−0.01913
D_5_	0	0

## Data Availability

The original contributions presented in this study are included in the article. Further inquiries can be directed to the corresponding author.
